# DNase γ Is the Effector Endonuclease for Internucleosomal DNA Fragmentation in Necrosis

**DOI:** 10.1371/journal.pone.0080223

**Published:** 2013-12-02

**Authors:** Ryushin Mizuta, Shinsuke Araki, Makoto Furukawa, Yuki Furukawa, Syota Ebara, Daisuke Shiokawa, Katsuhiko Hayashi, Sei-ichi Tanuma, Daisuke Kitamura

**Affiliations:** 1 Research Institute for Biomedical Sciences, Tokyo University of Science, Noda, Chiba, Japan; 2 Department of Biochemistry, Faculty of Pharmaceutical Sciences, Tokyo University of Science, Noda, Chiba, Japan; 3 Department of Molecular Immunology, Faculty of Pharmaceutical Sciences, Tokyo University of Science, Noda, Chiba, Japan; Juntendo University School of Medicine, Japan

## Abstract

Apoptosis and necrosis, two major forms of cell death, can be distinguished morphologically and biochemically. Internucleosomal DNA fragmentation (INDF) is a biochemical hallmark of apoptosis, and caspase-activated DNase (CAD), also known as DNA fragmentation factor 40 kDa (DFF40), is one of the major effector endonucleases. DNase γ, a Mg^2+^/Ca^2+^-dependent endonuclease, is also known to generate INDF but its role among other apoptosis-associated endonucleases in cell death is unclear. Here we show that (i) INDF occurs even during necrosis in cell lines, primary cells, and in tissues of mice *in vivo*, and (ii) DNase γ, but not CAD, is the effector endonuclease for INDF in cells undergoing necrosis. These results document a previously unappreciated role for INDF in necrosis and define its molecular basis.

## Introduction

Two cell death patterns, apoptosis and necrosis, can be distinguished from each other morphologically and biochemically [1], [2]. Typically necrosis is evoked by non-physiological stimuli that cause damage to the plasma membrane, and is characterized morphologically by cell swelling and lysis, and biochemically by random digestion of DNA, resulting in a smear on analysis by agarose gel electrophoresis. The necrosis-associated DNA degradation process and its physiological significance, however, have not been fully elucidated. Apoptosis, by contrast, is intrinsically programmed to eliminate undesirable cells during development, and to maintain homeostasis of the immune system [3], [4]. Apoptosis is characterized morphologically by the formation of membrane-associated apoptotic bodies, membrane blebbing, nuclear breakdown, and chromatin condensation, and biochemically by internucleosomal DNA fragmentation (INDF), appearing as a ladder on agarose gel electrophoresis. The DNA fragments expose 3′-OH ends, which can be detected microscopically as fluorescent signals using the terminal deoxynucleotidyl transferase (TdT)-mediated dUTP-biotin nick end labeling (TUNEL) assay. Thus, INDF, shown as DNA ladder and TUNEL, are widely accepted as the biochemical criterion of apoptosis.

Several endonucleases have been shown to be involved in apoptosis [4], [5]. Mg^2+^-dependent endonuclease CAD (also known as DFF40) is located in the nucleus, normally in an inactive complex with the chaperone ICAD (also known as DFF45) [6], [7]. Upon apoptosis-inducing stimuli, CAD is released from the complex by the caspase-3-mediated cleavage of ICAD and catalyzes INDF. In *CAD*-deficient mice, thymocytes do not undergo INDF [3]. Endonuclease G (Endo G), another Mg^2+^-dependent endonuclease [8], was shown to migrate from mitochondria into the nucleus to cleave chromosomal DNA. However, no apoptosis-related phenotype has been observed in *Endo G*-deficient mice [9]. DNase γ (also termed DNASE1L3) is Mg^2+^/Ca^2+^-dependent DNase I-family endonuclease that is located in the nuclear envelope and endoplasmic reticulum of living cells and translocates into the nucleus of dying cells, where it induces INDF [10]–[12]. DNase γ endonuclease activity is enhanced in the presence of linker histone H1, implicating its direct role in INDF [5]. DNase γ has been shown to be involved in apoptosis of a myoblastic cell line undergoing differentiation and of an immature B-cell line after signaling the antigen receptor [13], [14], but its *in vivo* role remains unknown.

In contrast to apoptotic endonucleases, little is known about the nucleases responsible for necrosis. Despite the general belief in the random nature of DNA degradation in necrosis, several studies have suggested that INDF can take place even in cells undergoing necrosis [1], [15]–[17]. Although caspase-independent DNase I and II are implicated in necrosis, their roles in INDF during necrosis are still unclear [2], [18]. In this study, we focused on DNase γ as a candidate endonuclease involved in INDF during necrosis.

## Results

### A selective role for DNase γ in necrosis-associated INDF

**Figure 1:**
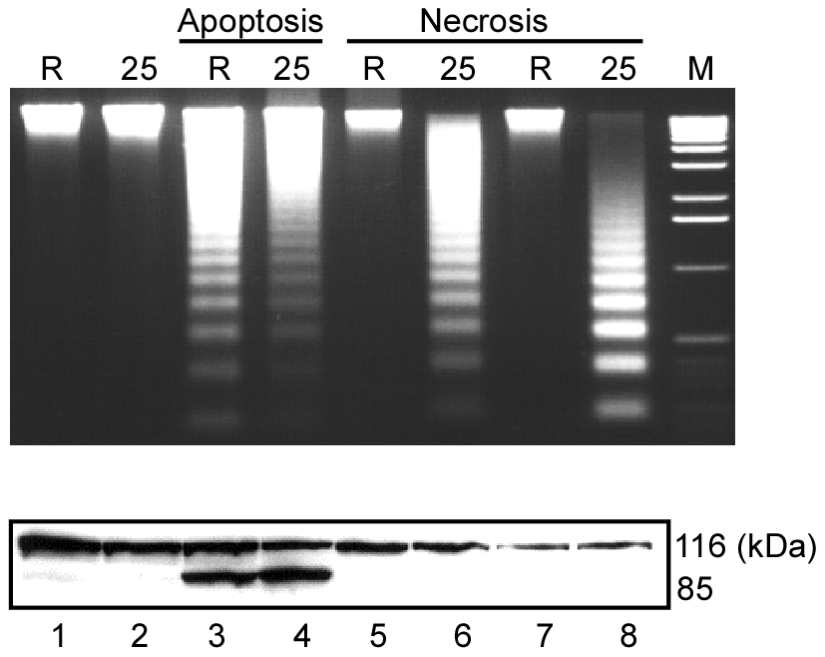
Direct comparison of DNase γ-dependence of DNA fragmentation in apoptosis and necrosis. Ramos (R) and γRamos-25 (25, a stable transformant over-expressing human DNase γ) cells were induced to undergo apoptosis with staurosporine (lanes 3 and 4) or necrosis by a freeze-thaw procedure (lanes 5 and 6) or saponin (lanes 7 and 8). DNA fragmentation analyzed by agarose-gel electrophoresis and PARP cleavage (from 116 kDa to 85 kDa) by western blot are shown at the top and bottom panels, respectively. Control DNA (top panel) and protein (bottom panel) from untreated cells are in lanes 1 and 2. M, 1-kb ladder DNA marker.

To directly compare the role of DNase γ in necrosis and apoptosis, we first used the Ramos Burkitt's lymphoma cell line, which has no DNase γ activity, and its derivative γRamos-25 stably expressing exogenous DNase γ [19]. Cells were treated with staurosporine to induce apoptosis [20], or by a freeze-thaw procedure or with saponin to induce necrosis [1]. Saponin treatment permeabilizes the plasma membrane as well as the membranes of organelles such as the endoplasmic reticulum and Golgi apparatus but not the nuclear membrane [1]. As shown in Figure 1, DNA ladders were detected in both Ramos and γRamos-25 after the staurosporine treatment (lanes 3 and 4), but only in γRamos-25 after treatment by freeze-thawing (lanes 5 and 6) or with saponin (lanes 7 and 8). The cleavage of poly-ADP ribose polymerase (PARP) from the 116-kDa to the 85-kDa form, a hallmark of apoptosis [20], was induced only by the staurosporine treatment (Fig. 1, bottom). This result suggests a selective role for DNase γ in the generation of INDF in necrosis.

### DNase γ mediates INDF in TNF-α mediated necrosis

**Figure 2:**
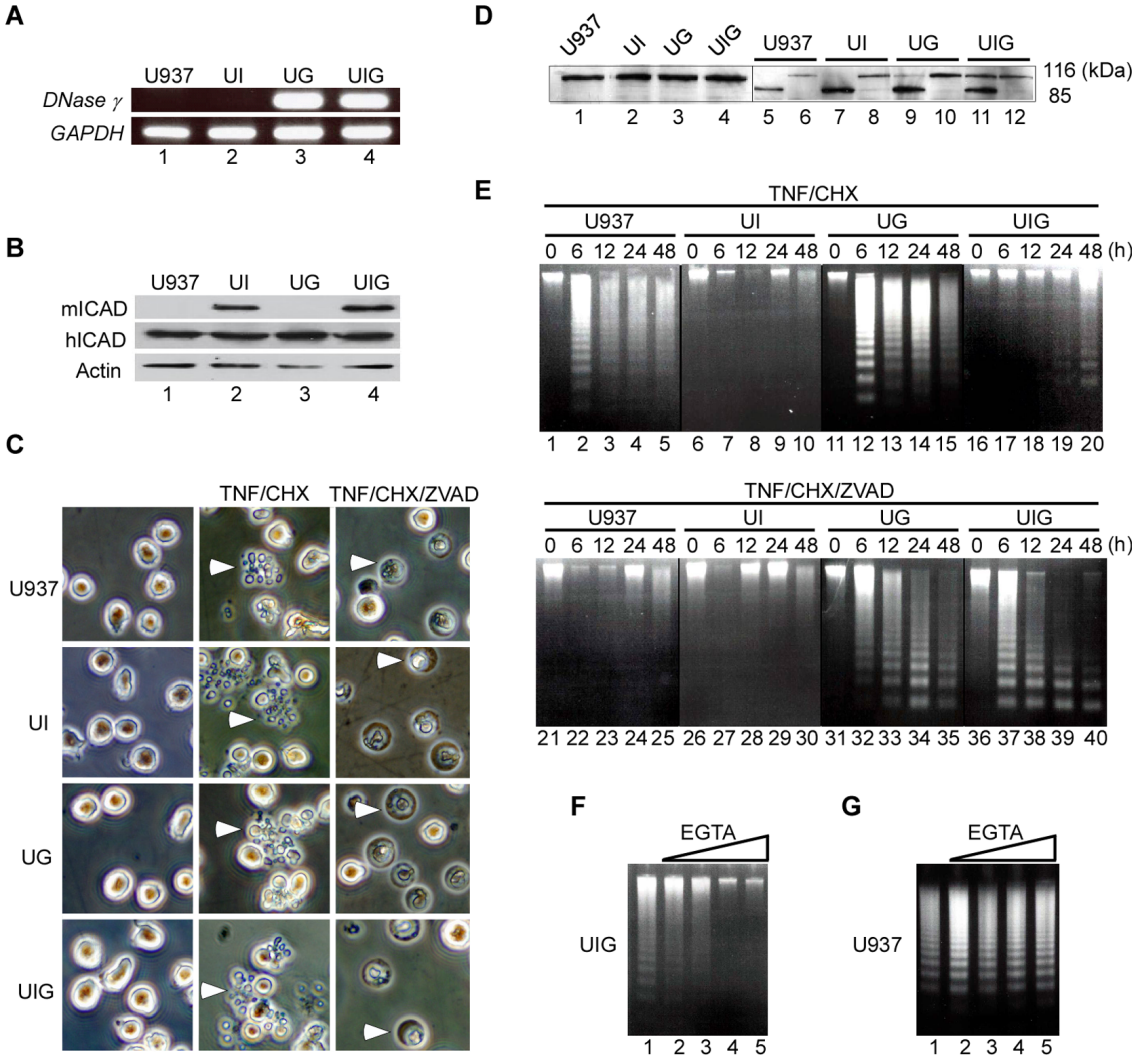
DNase γ-dependence of DNA fragmentation in TNF-receptor mediated necrosis. (**A**, **B**) Expression of DNase γ and ICAD in U937 and its derivatives (UI, UG, and UIG). (**A**) RT-PCR analysis of *DNase γ* and *GAPDH* expression. (**B**) Western blot detection of mouse ICAD-CR (mICAD), endogenous human ICAD (hICAD) and β-Actin. (**C**) Morphological changes in U937 cells and its derivatives undergoing apoptosis or necrosis. Apoptosis was induced with TNF-α and CHX (TNF/CHX, middle panels) and necrosis by additional pretreatment with Z-VAD-fmk (TNF/CHX/ZVAD, right panels). Cells were examined by phase contrast microscopy after 3 h incubation. Control untreated cells are shown in the left panels. Arrowheads in the middle and right panels indicate examples of cells with apoptotic bodies or swelling cells, respectively. (**D**) Western blot analysis showing PARP cleavage (from 116 kDa to 85 kDa). Lanes 1–4: untreated cells; Lanes 5, 7, 9, and 11: cells treated with TNF/CHX for 6 h; Lanes 6, 8, 10, and 12: cells treated with TNF/CHX/ZVAD for 6 h. (**E**) U937 and its derivatives were induced as in (**C**) and incubated for the indicated time periods (0–48 h). DNA samples were then examined by agarose gel electrophoresis. (**F**, **G**) Ca^2+^ is required for necrotic but not apoptotic DNA fragmentation. Necrosis and apoptosis were induced in UIG with TNF/CHX/ZVAD for 6 h (**F**) and U937 with TNF/CHX for 6 h (**G**), respectively, in the presence of various concentrations of EGTA (lanes 1–5; 0 mM, 0.25 mM, 0.5 mM, 1 mM, and 2 mM). After the incubation, DNA from each sample was analyzed by agarose gel electrophoresis. Data shown are representative of two to three separate experiments.

Next we utilized the U937 human hystiocytic leukemia cell line, which is known to undergo apoptosis upon treatment with TNF-α in the presence of cycloheximide (CHX), but necrosis in the additional presence of Z-VAD-fmk, a pan-caspase inhibitor [21]. Our stock of U937 cells did not express detectable *DNase γ* mRNA, therefore we generated three U937 stable transformants: UI, expressing a caspase-resistant mutant of mouse ICAD (ICAD-CR) as a dominant inhibitor of CAD [22]; UG, expressing DNase γ; and UIG, expressing both ICAD-CR and DNase γ (Fig. 2A and 2B). After treatment with TNF-α and CHX (TNF-α/CHX), all four cell lines underwent apoptosis as evident from the appearance of apoptotic bodies (Fig. 2C, center panels) and the diagnostic cleavage of PARP (Fig. 2D, lanes 5, 7, 9, and 11). In the additional presence of Z-VAD-fmk (TNF-α/CHX/ZVAD), however, the cells showed no evidence of apoptosis phenotype, but instead underwent necrosis, as is evident from cell swelling (Fig. 2C, right panels) and the absence of PARP cleavage (Fig. 2D, lanes 6, 8, 10, and 12). Under conditions leading to apoptosis (TNF-α/CHX), DNA ladders were detected in U937 and UG cells after 6 h of incubation, but were hardly detected in UI and UIG cells (Fig. 2E, top), indicating the CAD-dependent generation of INDF. Weak ladder formation in UIG cells only after 24 h of incubation suggests that a contribution of DNase γ to INDF occurs only at the terminal stage of apoptosis (lanes 19 and 20). On the other hand, under conditions causing necrosis (TNF-α/CHX/ZVAD), DNA ladders were formed only in UG and UIG, beginning as early as 6 h of incubation (Fig. 2E, bottom). Thus, the generation of INDF during necrosis depends on DNase γ, but not on CAD activity. This conclusion was further supported by the observation that chelating Ca^2+^ with EGTA inhibited DNA ladder formation in UIG cells undergoing necrosis, but not in U937 cells undergoing apoptosis (Fig. 2F and 2G), since DNase γ, but not CAD, requires Ca^2+^ for its endonuclease activity. The terminal phase of apoptosis where DNase γ–mediated DNA laddering appears (Fig. 2E, top, lanes 19 and 20) may be regarded as secondary necrosis, since plasma membrane permeabilization should be inevitable at this stage [23].

### DNase γ generates TUNEL signals in necrotic cells

**Figure 3:**
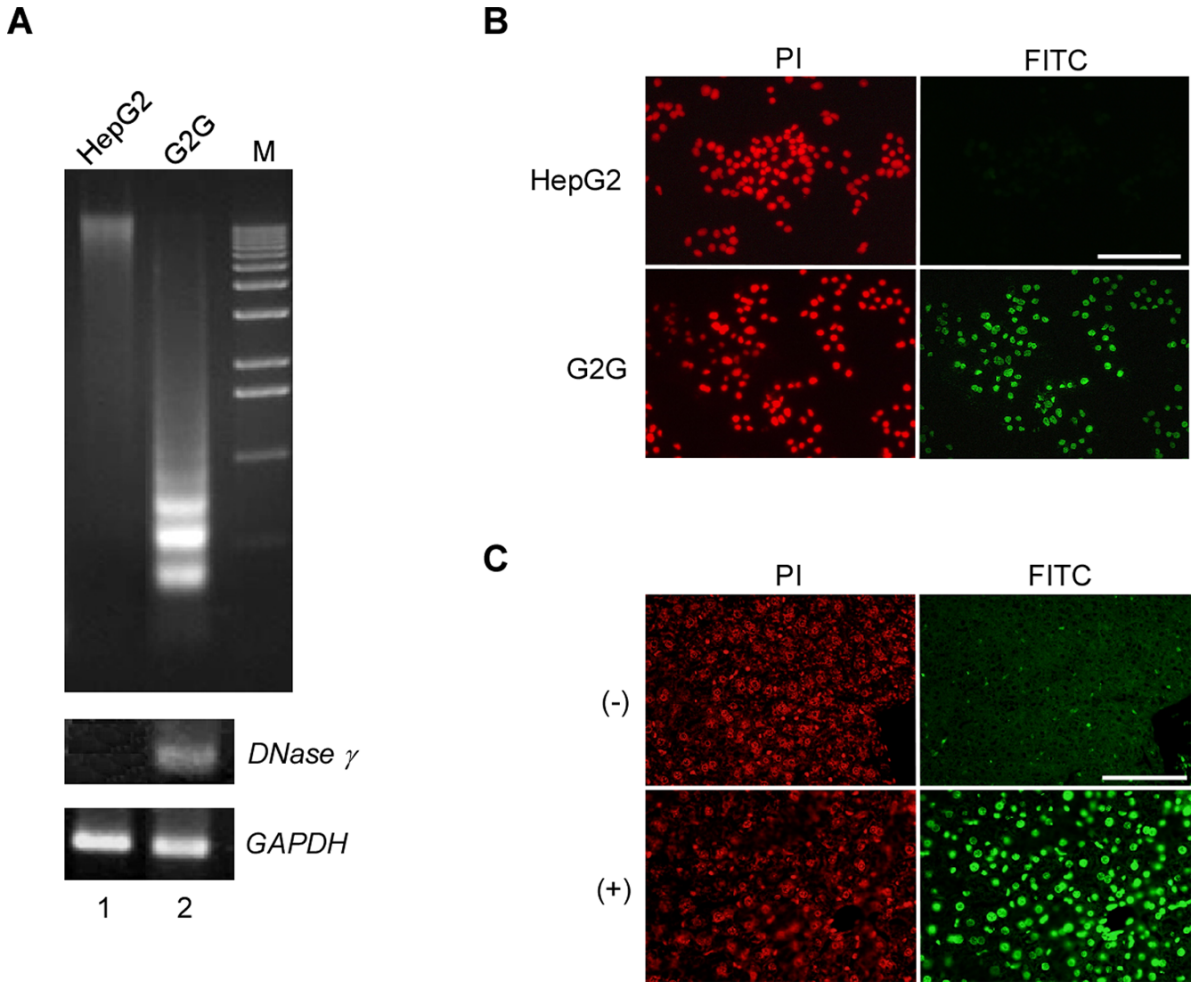
DNase γ is responsible for generation of TUNEL-positive DNA ends in necrotic cells. (**A**) HepG2 and G2G cells were treated with digitonin for 1 h, and genomic DNAs from these cells were electrophoresed on agarose gels (top panel): M, 1-kb ladder DNA marker. *DNase γ* and *GAPDH* expression levels in the same cells were examined by RT-PCR assay (middle and bottom panels). (**B**) HepG2 and G2G cells were treated with digitonin for 30 m and subjected to TUNEL assay (right panels) and stained with propidium iodide (PI, left panels). (**C**) Fixed mouse liver sections were treated with (+, bottom panels) or without (−, top panels) recombinant DNase γ (2.5 ng/ml) at 37°C for 30 min and subjected to TUNEL assay (right panels) and stained with PI (left panels). PI stained both DNA and RNA. Scale bar, 200 µm.

As mentioned above, the TUNEL assay was originally developed to identify apoptotic cells by detecting the free 3′-OH DNA ends, but it has been reported that necrotic cells are also detectable by this assay [15], [17]. Since DNase γ is known to generate 3′-OH DNA ends [24], we examined whether DNase γ is responsible for the TUNEL signals seen in necrotic cells. HepG2, a human hepatocellular carcinoma cell line that does not express DNase γ, and its derivative expressing exogenous DNase γ (G2G, Fig. 3A) were treated with the detergent digitonin to induce necrosis [25]. Not only DNA ladder formation but also TUNEL signals were detected in G2G cells but not in HepG2 cells (Fig. 3A and 3B). In addition, when fixed mouse liver sections were treated with recombinant DNase γ protein, the liver cell nuclei became TUNEL-positive (Fig. 3C). These data indicate that DNase γ is responsible for TUNEL signals generated in necrotic cells.

### Necrosis-associated INDF is abrogated in *DNase γ*-deficient mice

To investigate the function of DNase γ *in vivo*, we generated *DNase γ*-deficient mice through gene targeting, in which codon His160, comprising the catalytic center of the endonuclease, was replaced with a selectable marker gene (Fig. S1A). Homologous recombination in the *DNase γ* gene locus was confirmed by Southern blot analysis (Fig. S1B), and disruption of DNase γ activity was confirmed by zymography (Fig. S1C). Homozygous *DNase γ* ^−/−^ mice were born at the expected Mendelian ratio and did not show any gross abnormalities.

**Figure 4:**
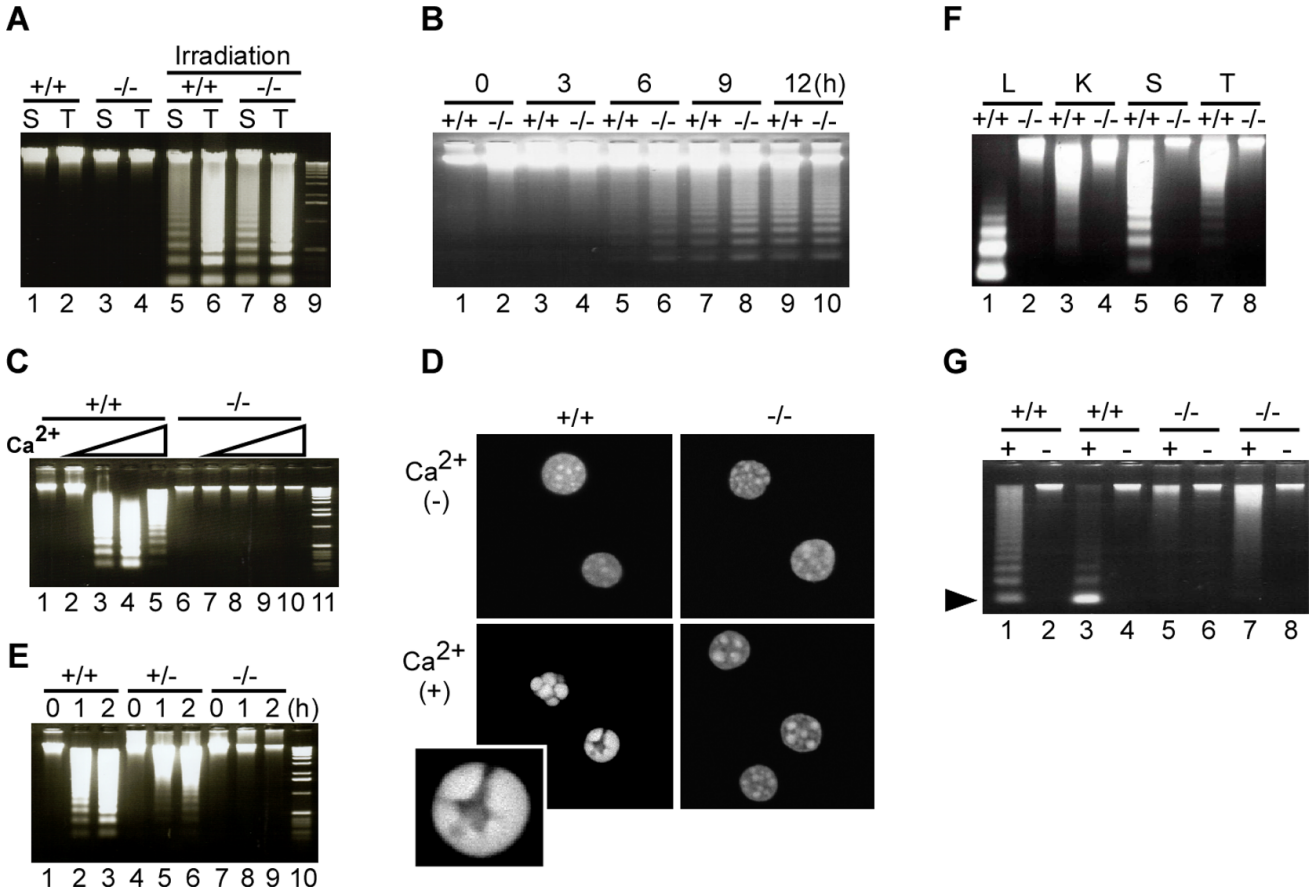
Necrosis-associated DNA fragmentation is abrogated in *DNase γ*-deficient mice. (**A–C**, **E–G**) Fragmentation of genomic DNA was analyzed by agarose gel electrophoresis. (**A**) Apoptosis was induced in *DNase γ* ^+/+^ (+/+) and *DNase γ* ^−/−^ (−/−) mice by γ-irradiation. Genomic DNA from spleen (S) or thymus (T) was analyzed (lanes 5–8). Lanes 1–4, DNA from non-irradiated mice. Lane 9, 1-kb ladder. (**B**) Primary hepatocytes from mice of the indicated genotypes were incubated at 37°C with anti-Fas antibody for the indicated time periods (0–12 h) and DNA fragmentation was analyzed. (**C**) Hepatocyte nuclei were incubated at 37°C for 2 h in buffer containing 5 mM Mg^2+^ and various concentrations of Ca^2+^ (lanes 1 and 6, 0 mM; lanes 2 and 7, 0.3 mM; lanes 3 and 8, 1 mM; lanes 4 and 9, 5 mM; lanes 5 and 10, 10 mM), and DNA was analyzed. Lane 11, 1-kb ladder. (**D**) The same nuclei as in (**C**) were incubated for 2 h in buffer with 5 mM Mg^2+^ and with or without 5 mM Ca^2+^ (bottom and top panels, respectively), stained with 4′,6-Diamidine-2′-phenylindole dihydrochloride (DAPI) and examined by confocal microscopy. Magnification, ×600. A high power image of chromatin condensation is shown in the inset. (**E**) Splenocytes from mice of the indicated genotypes were incubated with saponin in DMEM at 37°C for the indicated time periods (0–2 h) to induce necrosis, and DNA from these cells was analyzed. Lane 10, 1-kb ladder. (**F**) Frozen tissues (L: liver; K: kidney; S: spleen; T: thymus) from mice of the indicated genotypes were smashed to a homogenate, incubated in DMEM at 37°C for 1 h and then used for DNA purification. (**G**) DNA fragmentation in liver after ischemia-induced necrosis. Ischemia was induced in one of the hepatic lobes of two mice from each genotype and DNA was prepared from the ischemic (+) and nonischemic lobes (−). Arrowhead, 180 bp DNA fragments corresponding to a single nucleosome.

*DNase γ* ^+/+^ and *DNase γ* ^−/−^ mice were γ-ray irradiated to induce apoptosis *in vivo* [26]. Spleen and thymic DNA from both strains displayed comparable DNA laddering when examined by agarose gel electrophoresis (Fig. 4A). Anti-Fas antibody induces apoptosis of cultured primary hepatocytes [27], and again the DNA ladder formation was indistinguishable between *DNase γ* ^+/+^ and *DNase γ* ^−/−^ mice (Fig. 4B). However, when the isolated hepatocyte nuclei were incubated in the presence of Mg^2+^ (5 mM) and 1–10 mM Ca^2+^, DNA from the nuclei of *DNase γ* ^+/+^ mice exhibited DNA ladders but those of *DNase γ* ^−/−^ mice did not at any Ca^2+^ concentration (Fig. 4C). The nuclei of *DNase γ* ^+/+^, but not *DNase γ* ^−/−^ mice, showed chromatin condensation visible by fluorescence confocal microscope after incubation with Mg^2+^ and Ca^2+^ (Fig. 4D). These data indicate that the increase of Ca^2+^ in the nucleus induces INDF and chromatin condensation in a DNase γ-dependent manner. It was previously reported that Mg^2+^/Ca^2+^-dependent chromosomal DNA breaks occur in necrotic cells permeabilized with mild detergents such as saponin [28], suggesting a role for DNase γ. Indeed, splenocytes of *DNase γ* ^+/+^ and *DNase γ* ^+/−^, but not *DNase γ* ^−/−^ mice, underwent DNA laddering soon after incubation with saponin in the presence of Mg^2+^ and Ca^2+^ (Fig. 4E). We then induced necrosis in the bulk of tissues excised from both *DNase γ* ^+/+^ and *DNase γ* ^−/−^ mice by rapid freezing and mechanical smashing, followed by incubation in medium. As shown in Figure 4F, DNA from the liver, kidney, spleen, and thymus of *DNase γ* ^+/+^ mice exhibited DNA ladders, but that from *DNase γ* ^−/−^ mice did not.

Finally, we examined the role of DNase γ in necrosis occurring *in vivo* using an ischemia-induced cell death model [29]. We induced liver ischemia by ligating a portion of one hepatic lobe for 8 h in live animals. DNA was extracted from the ligated and nonligated lobes from *DNase γ* ^+/+^ and *DNase γ* ^−/−^ mice and electrophoresed on agarose gels (Fig. 4G). As expected, DNA from the nonligated lobes did not show any DNA ladders or smears (lanes 2, 4, 6, and 8). DNA samples from the ligated lobes of *DNase γ* ^+/+^ mice showed DNA ladders (lanes 1 and 3), whereas those of *DNase γ* ^−/−^ mice did not show ladders but only smears (lanes 5 and 7). Thus, DNase γ is essential for INDF generated during necrosis of ischemic liver cells *in vivo*. The random DNA degradation detected as smears in the liver of the *DNase γ* ^−/−^ mice is likely caused by other ubiquitous endonucleases such as DNase I. Altogether, these results indicate that INDF occurring during necrosis is carried out by DNase γ both *in vitro* and *in vivo*.

## Discussion

DNA ladders and TUNEL signals, the major hallmarks of INDF, are well known features of apoptosis. Although they have occasionally been detected in cells undergoing necrosis [1], [15]–[17], this outcome has not been widely appreciated and a DNA smear caused by random DNA degradation has rather been considered to be a common feature of necrotic DNA degradation. This is partly because the molecular mechanisms of necrosis have been poorly understood. Here, we showed that DNase γ is responsible for INDF during necrosis induced by TNF-α, plasma membrane disruption, or freeze-thawing of cells *in vitro* and in ischemic tissues *in vivo*. These results thus establish that (i) INDF is a feature of DNA degradation during necrosis and (ii) it is carried out by DNase γ. There may be several reasons why DNA ladders are not commonly observed in necrosis, including late timing of sampling and analysis and also the presence of a dominant population of intact cells in some conditions that induce necrosis. In this study, we used the Wizard DNA purification resin for DNA preparation. This procedure is helpful for detecting DNA ladder, because the resin selectively recovers and concentrates low-molecular weight DNA fragments. We also detected chromatin condensation under necrotic condition in a DNase γ-dependent manner (Fig. 4D). Chromatin condensation is also known as a hallmark of apoptosis but the molecular mechanism has not been clarified [30]. Thus, the mechanism and physiological significance of DNase γ-dependent chromatin condensation should be examined in further study.

DNase γ is normally found in the nuclear envelope and endoplasmic reticulum, but is localized in the nucleus of dying cells [24]. Based on our data, we propose the following possible scenarios for the role of DNase γ in necrosis: In cells undergoing necrosis, the disruption of the plasma and organelle membranes causes Ca^2+^ influx into the nucleus, leading to activation of DNase γ diffused from the nuclear envelope and endoplasmic reticulum, and the DNase γ then executes INDF. Alternatively, DNase γ may be secreted, as previously reported [31], diffusing into necrotic cells together with Ca^2+^ through the damaged plasma membrane to the nucleus. Either scenario would explain the ladder formation in the terminal phase of apoptosis, often called secondary necrosis, even in the absence of CAD activity (Fig. 2E) [23]. In the case of apoptosis, DNase γ is not necessarily responsible for INDF (e.g. Fig. 1, Fig. 2E), and CAD is responsible. Thus, INDF is likely a common outcome of dying cells, but it is differentially processed in apoptosis vs. necrosis, for the latter uniquely by DNase γ.

As for the physiological significance of INDF in cell death, strong evidence that DNA degradation is important for avoiding unwanted immune activation has come from studies of mice that are deficient in endonucleases [32]–[34]. In phagocytes, DNase II inside the phagocytic lysosomal organelles digests DNA of engulfed apoptotic cells. Deficiency in DNase II results in accumulation of DNA in these organelles, which stimulates the pro-inflammatory activity of phagocytes [32]. DNase I digests the DNA of the chromatin released into the extracellular space after cell death. DNase-I-deficient mice produce autoantibodies and manifest lupus-like autoimmune diseases [33]. Thus the physiological significance of INDF might be to render the chromatin more manageable for subsequent disposal by phagocytic cells. The specific role of DNase γ–dependent necrotic DNA fragmentation *in vivo* has not yet been addressed because a condition causing local necrosis without lethal damage is difficult to achieve. We also have not detected any gross abnormalities in DNase-γ-deficient mice. More specific functions, especially immune functions, should be examined in further study.

In this paper we showed that DNase γ carries out INDF in cells undergoing necrosis. This finding should have a substantial impact on studies of cell death, because INDF has been widely accepted for a long time as the hallmark of apoptosis. Our studies clearly suggest that this criterion is not definitive, since even when INDF is detected, it is not always an indicator of apoptosis, and DNase γ-related necrosis should also be considered.

## Methods

### Ethics Statement

All experimental procedures using mice were approved by the Institutional Animal Care and Use Committee at the Tokyo University of Science.

### Reagents and animals

The following reagents were obtained commercially: Staurosporine (Calbiochem); 4′,6-Diamidine-2′-phenylindole dihydrochloride (DAPI) (Boheringer Mannheim); saponin, digitonin, actinomycin D and CHX (Sigma-Aldrich); Proteinase K (MERCK); TNF-α, anti-PARP antibody (Ab), and guanidine hydrochloride (Wako); anti-mouse ICAD Ab (eBioscience); anti-actin Ab (Santa Cruz); anti-Fas Ab (Jo2) and anti-human ICAD (DFF45) Ab (BD Pharmingen). C57BL/6 mice were purchased from Sankyo-Lab Service (Tsukuba, Japan).

### Cells and plasmid constructs

Ramos and γRamos-25, a stable transformant over-expressing human DNase γ, were described previously [19]. U937 cells were transfected with the expression vectors encoding caspase-resistant mouse ICAD (ICAD-CR) [22], pCAG/ICAD-CR-Myc-His, or human DNase γ [19], phDNase γ-Myc-His, and selected with puromycin (1 µg/ml) or G418 (2 mg/ml), respectively, to generate stable clones (UI, UG, and UIG). HepG2 cells were transfected with phDNase γ-Myc-His (puro), a puromycin resistant version of phDNase γ-Myc-His, and selected with puromycin (2 µg/ml) to generate a stable clone (G2G). Cells were cultured in RPMI1640 medium or DMEM supplemented with 10% fetal bovine serum and 100 U/ml penicillin–streptomycin. Mouse spleen cells were freed of red blood cells and dead cells using Lympholyte (Cedarlane) according to the supplier's protocol. Mouse parenchymal hepatocytes were isolated by *in situ* collagenase perfusion as described previously [35].

### DNA preparation

For the DNA ladder formation assay, cells (0.5–1×10^6^) were harvested by centrifugation, suspended in 60 µl of PBS and lysed by addition of 300 µl of 7 M guanidine. DNA was isolated from the lysate with the Wizard DNA purification resin (Promega) as described [12]. To prepare DNA from primary hepatocytes, harvested cells were lysed in lysis buffer (100 mM Tris-HCl pH 8.5, 5 mM EDTA, 0.2% Sodium Dodecyl Sulfate [SDS], 200 mM NaCl, 100 µg/ml Proteinase K) at 55°C overnight and DNA was precipitated with isopropanol. To prepare DNA from tissues, tissue samples were homogenized with the BioMasher tissue homogenizer (Nippi) and the homogenates (5 mg) were suspended in 60 µl of PBS or DMEM. DNA was prepared from this diluted homogenate with the Wizard DNA purification resin. DNA was analyzed by electrophoresis in a 1.5% agarose gel in the presence of ethidium bromide (0.5 µg/ml).

### DNA fragmentation and chromatin condensation of hepatocyte nuclei

Mouse liver nuclei were prepared as previously described [12]. The reaction mixture for the DNA fragmentation assay contained 25 mM Tris-HCl (pH 7.4), 150 mM KCl, 5 mM MgCl_2_, various concentrations of CaCl_2_ and 5×10^5^ nuclei in a final volume of 60 µl. The reaction was performed at 37°C for 2 h, after which DNA was isolated from the nuclei using the Wizard DNA purification resin as described above. Portions of the nuclear incubation mixtures containing 0 mM or 5 mM CaCl_2_ were centrifuged in a microfuge at 10,000 r.p.m. for 2 min and the nuclei in the resulting pellets were resuspended in the fixation solution (4% formaldehyde in PBS) and stained with DAPI. The nuclei were then examined with a confocal microscope (Leica, TCS SP2).

### Cell death induction

To induce apoptosis in cultured cells, Ramos and γRamos-25 cells were incubated with staurosporine (1 µM) at 37°C for 6 h, and U937 and its derivatives were incubated with TNF-α (5 ng/ml) and CHX (1 µg/ml) at 37°C for the indicated time. To induce hepatocyte apoptosis, primary hepatocytes (0.5×10^6^) were cultured with anti-Fas antibody (0.5 µg/ml) and actinomycin D (0.05 µg/ml). To induce apoptosis *in vivo*, mice were γ-ray irradiated (12 Gy) and were sacrificed after 4 h. Spleen and thymus were excised and the DNA was prepared as above. To induce necrosis by freeze-thawing, cells (0.5–1×10^6^) were harvested by centrifugation and the cell pellets were frozen in a deep freezer (−80°C). The cells were then thawed, suspended in 120–180 µl of DMEM supplemented with 25 mM Hepes (DMEM/Hepes), and incubated at 37°C for the indicated times. To induce necrosis with saponin or digitonin, cells (0.5–1×10^6^) were harvested and suspended in 120–180 µl of DMEM/Hepes. Saponin or digitonin were added to the cell suspension (0.2 µg/ml or 40 µg/ml, respectively) and incubated at 37°C for 1 h. To induce death-receptor mediated necrosis, cells were treated with Z-VAD-fmk (20 µM) 30 min before challenge of TNF-α (5 ng/ml) and CHX (1 µg/ml). Some cells were incubated with EGTA (0.25–2 mM) to chelate calcium. Cells were incubated for the indicated times, examined by microscopy and harvested for DNA preparation. To induce tissue necrosis, excised tissues were immediately frozen in liquid nitrogen and homogenized with the BioMasher tissue homogenizer (Nippi). The smashed homogenate (5 mg) was incubated in 60 µl of DMEM/Hepes at 37°C for 1 h and DNA was prepared from the sample with the Wizard DNA purification resin.

### Ischemia induction *in vivo*

Mice were anesthetized by tribromoethanol. After midline laparotomy, a part of one hepatic lobe was ligated with 4-0 silk sutures. The abdomen was closed in layers, and the animals were allowed to recover. The animals were sacrificed after 8 h by cervical dislocation. The ligated ischemic and non-ligated healthy lobes were excised from the mice and DNA was prepared by the Wizard DNA purification resin after homogenizing the tissues with the BioMasher tissue homogenizer as above.

### Western blot analysis

Cells (1×10^6^) were harvested, suspended in 50 µl of PBS and lysed in 50 µl of sample buffer (4% SDS, 100 mM Tris-HCl pH 6.8, 2 mg/ml Bromophenol Blue [BPB], 20% glycerol, 200 mM DTT). The samples were separated by SDS-PAGE with a 10% gel and transferred to a nylon membrane. The membrane was incubated with the indicated primary antibody and the appropriate HRP-conjugated secondary antibody. Signals were visualized with the ECL kit (Amersham).

### TUNEL assay

HepG2 and G2G cells were plated on coverslips and cultured overnight in DMEM medium. Then the cells were permeabilized with digitonin (40 µg/ml) to induce necrosis and incubated in DMEM/Hepes at 37°C for 30 min, after that the cells were fixed with 4% formalin. Excised liver was fixed in 4% formalin and embedded in paraffin. Four micron liver sections were deparaffinized and incubated in DNase γ reaction solution (DMEM/Hepes) with recombinant DNase γ (2.5 ng/ml) at 37°C for 30 min. The cleaved DNA ends were stained using the DeadEnd Fluorometric TUNEL System (Promega) according to the manufacturer's protocol. The samples were also stained with propidium iodide and examined by microscopy (KEYENCE, BZ-9000).

### Extraction of total RNA and RT-PCR

Total RNA was extracted from cells using TRIzol (Sigma-Aldrich) according to the manufacturer's instructions. Ten micrograms of total RNA was reverse-transcribed into cDNA using Superscript II with oligo(dT) primers (Invitrogen). Thereafter, the resultant cDNA was amplified together with GoTaq DNA polymerase (Promega) using gene-specific primer pairs: DNase γ sense, 5′-GAAGGTCATCAAACGCTGTG-3′; DNase γ antisense, 5′-GGAACCAGACCACAAAG-3′; GAPDH sense, 5′-TCCACCACCCTGTTGCTGTAG-3′; GAPDH antisense, 5′-GACCACAGTCCATGCCATCACT-3′. The amplified PCR products were fractionated on 1.5% agarose gels and visualized by ethidium bromide staining.

### Establishment of *DNase γ* knockout ES cells and mice

A DNA fragment containing the mouse *DNase γ* gene was isolated from a C57BL/6 genomic DNA library (Stratagene). Using this genomic DNA clone as a template, a 4.9-kb DNA fragment, corresponding to intron 4 and part of exon 5 of the *DNase γ* gene, was amplified by PCR with the following primers: DNaseF8, 5′-GACGTCGACCCCCAACAACTTGGCTATGGGTCC-3′, and DNaseR4, 5′-CTCACTAGTGGGGACAATCACGAAGTCCTTGAC-3′. This 4.9-kb fragment and the 6.0-kb Acc I fragment spanning from exon 5 to intron 6 of the *DNase γ* gene were used for constructing the gene-targeting vector. In the resulting targeting vector, pLNTK-γ-KO3, the sequence of 17 amino acids (Leu159-Asp175) including the active site residue (His160) of the mouse DNase γ protein (NCBI accession no. AAH12671) is deleted from exon 5. After linearization with *Sal I*, the targeting vector was transfected into 3×10^7^ ES cells (Bruce 4, C57BL/6 origin) [36], and the targeted clones were selected with 0.2 mg/ml of G418 and 2 µM of ganciclovir. The drug-resistant colonies were expanded and the homologous recombination event was confirmed by Southern blot analysis (described below). The targeted ES cell clones were used for the generation of chimeric mice. Heterozygous (*DNase γ* ^+/−^) and then the homozygous mice (*DNase γ* ^−/−^) of C57BL/6 background were generated from these chimeric mice.

### Southern blot analysis

Genomic DNA (10 µg) was digested with *Hind III* or *Spe I* and *Kpn I*, separated by 0.8% agarose-Tris-acetate-EDTA gel electrophoresis, transblotted to a nylon membrane, and probed with the ^32^P-labeled DNA fragments located outside of the targeting vector (either 5′- or 3′- probes, respectively). The 5′- and 3′- probes were generated by PCR using genomic DNA as a template with two primer pairs, DNaseF5-DNaseR3 and mGSP5-HA1, respectively. (DNaseF5, 5′-CCTTGTCAACAACAACCACC-3′; DNaseR3, 5′-ACCATGCCTTGCCTAGAATC-3′; mGSP5, 5′-AGTCAACTCCGTGGTTCCCCGTTCC-3′; HA1, 5′-GTGATCACTGACATCCAGGG-3′)

### Activity gel analysis

The activity gel system (Zymography) was used for the identification of DNase activity [37]. Briefly, protein extracts from spleen cells were electrophoresed in a Laemmli SDS gel containing salmon sperm DNA (200 µg/ml). After washing, the gel was incubated in a reaction buffer (10 mM Tris-HCl [pH 7.8], 3 mM CaCl_2_ and 3 mM MgCl_2_), at 37°C for 24 h. DNase activity was detected as an ethidium bromide-unstained dark band under ultraviolet illumination.

## Supporting Information

Figure S1 **Disruption of DNase γ function by gene targeting.** (**A**) Schematic representation of the wild type *DNase γ* allele, the targeting construct and the targeted allele. Exons (with numbers) and introns are indicated by open boxes and horizontal lines, respectively. The neomycin resistance gene (NEO) and the herpes simplex virus thymidine kinase gene (HSV-TK) are indicated. 5′- and 3′-probes used in Southern blot analysis are shown by closed boxes. Restriction fragments detected by these probes are shown by double-headed arrows. A codon for the catalytic residue (His 160) in exon 5 of the mouse *DNase γ* gene was disrupted by the replacement with Neo. (**B**) Southern blot analysis of genomic DNA from tails of wild-type (+/+), heterozygous (+/−) and homozygous (−/−) *DNase γ* mice. Left: genomic DNA was digested with *Hind III* and hybridized with the 5′-probe. Right: genomic DNA was digested with *Spe I* and *Kpn I* and hybridized with the 3′-probe. (**C**) DNase γ activity gel assay. Splenocyte-nuclear extracts from the indicated mice were subjected to DNase γ activity gel assay. The activity was detected as dark areas on fluorescent background by the UV transillumination of the gel. DNase activity was detected in wild-type (+/+) and heterozygous (+/−) *DNase γ* mice, but not in homozygous (−/−) *DNase γ* mice.
(DOC)
