# Supplementary material for: DNase γ Is the Effector Endonuclease for Internucleosomal DNA Fragmentation in Necrosis
Source: PLoS One. 2013 Dec 2;8(12):e80223. doi: 10.1371/journal.pone.0080223 (PMC3846476; doi:10.1371/journal.pone.0080223)
Supplement: Figure S1 — Disruption of DNase γ function by gene targeting. (A) Schematic representation of the wild type DNase γ allele, the targeting construct and the targeted allele. Exons (with numbers) and introns are indicated by open boxes and horizontal lines, respectively. The neomycin resistance gene (NEO) and the herpes simplex virus thymidine kinase gene (HSV-TK) are indicated. 5′- and 3′-probes used in Southern blot analysis are shown by closed boxes. Restriction fragments detected by these probes are shown by double-headed arrows. A codon for the catalytic residue (His 160) in exon 5 of the mouse DNase γ gene was disrupted by the replacement with Neo. (B) Southern blot analysis of genomic DNA from tails of wild-type (+/+), heterozygous (+/−) and homozygous (−/−) DNase γ mice. Left: genomic DNA was digested with Hind III and hybridized with the 5′-probe. Right: genomic DNA was digested with Spe I and Kpn I and hybridized with the 3′-probe. (C) DNase γ activity gel assay. Splenocyte-nuclear extracts from the indicated mice were subjected to DNase γ activity gel assay. The activity was detected as dark areas on fluorescent background by the UV transillumination of the gel. DNase activity was detected in wild-type (+/+) and heterozygous (+/−) DNase γ mice, but not in homozygous (−/−) DNase γ mice. (DOC) [file pone.0080223.s001.doc]

Supporting information

For:

DNase γ is the Effector Endonuclease for Internucleosomal DNA Fragmentation in Necrosis

**Ryushin Mizuta^1^*****, Shinsuke Araki^1, 2^, Makoto Furukawa^1^, Yuki Furukawa^1^, Syota Ebara^1^, Daisuke Shiokawa^2 †^, Katsuhiko Hayashi^1 ‡^, Sei-ichi Tanuma^2^, Daisuke Kitamura^1, 3^***

^1^Research Institute for Biomedical Sciences, Tokyo University of Science, 2669 Yamazaki, Noda, Chiba 278-0022, Japan.

^2^Department of Biochemistry, Faculty of Pharmaceutical Sciences, Tokyo University of Science, 2669 Yamazaki, Noda, Chiba 278-0022, Japan.

^3^Department of Molecular Immunology, Faculty of Pharmaceutical Sciences, Tokyo University of Science, 2669 Yamazaki, Noda, Chiba 278-0022, Japan.

*Correspondence to: Ryushin Mizuta, mizuta@rs.noda.tus.ac.jp; Daisuke Kitamura, kitamura@rs.noda.tus.ac.jp.


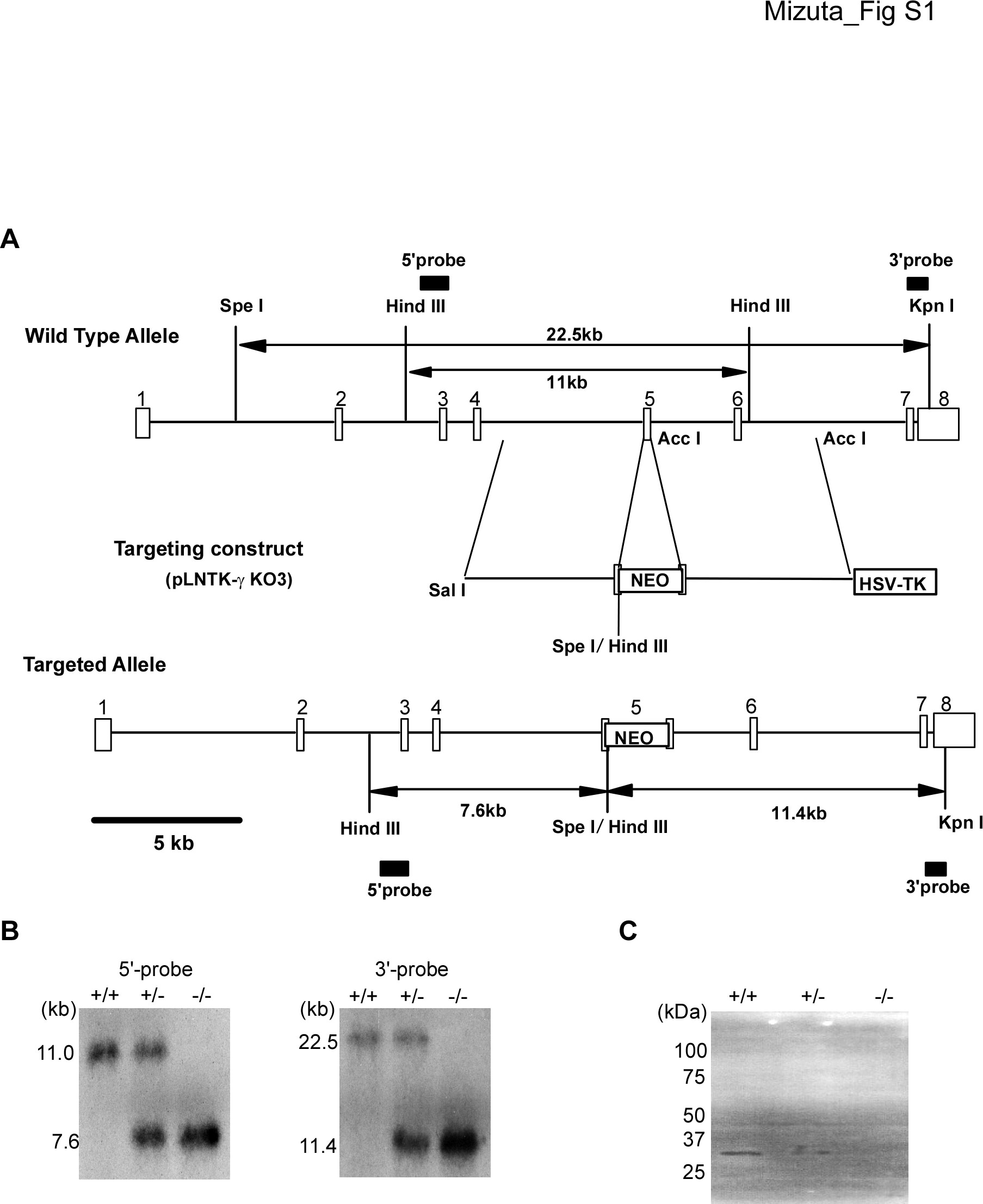


**Figure S1.** **Disruption of DNase γ function by gene targeting.** (**A**) Schematic representation of the wild type *DNase γ* allele, the targeting construct and the targeted allele. Exons (with numbers) and introns are indicated by open boxes and horizontal lines, respectively. The neomycin resistance gene (NEO) and the herpes simplex virus thymidine kinase gene (HSV-TK) are indicated. 5’- and 3’-probes used in Southern blot analysis are shown by closed boxes. Restriction fragments detected by these probes are shown by double-headed arrows. A codon for the catalytic residue (His 160) in exon 5 of the mouse *DNase γ* gene was disrupted by the replacement with Neo. (**B**) Southern blot analysis of genomic DNA from tails of wild-type (+/+), heterozygous (+/-) and homozygous (-/-) *DNase γ* mice. Left: genomic DNA was digested with *Hind III* and hybridized with the 5’-probe. Right: genomic DNA was digested with *Spe I* and *Kpn I* and hybridized with the 3’-probe. (**C**) DNase γ activity gel assay. Splenocyte-nuclear extracts from the indicated mice were subjected to DNase γ activity gel assay. The activity was detected as dark areas on fluorescent background by the UV transillumination of the gel. DNase activity was detected in wild-type (+/+) and heterozygous (+/-) *DNase γ* mice, but not in homozygous (-/-) *DNase γ* mice.
